# In Vitro Digestion and Storage Stability of β-Carotene-Loaded Nanoemulsion Stabilized by Soy Protein Isolate (SPI)-Citrus Pectin (CP) Complex/Conjugate Prepared with Ultrasound

**DOI:** 10.3390/foods11162410

**Published:** 2022-08-11

**Authors:** Xiaobin Ma, Tianyi Yan, Song Miao, Like Mao, Donghong Liu

**Affiliations:** 1 Teagasc Food Research Centre, Moorepark, Fermoy, P61 C996 Cork, Ireland; 2 Zhejiang R&D Center for Food Technology and Equipment, National-Local Joint Engineering Laboratory of Intelligent Food Technology and Equipment, Zhejiang Key Laboratory for Agro-Food Processing, College of Biosystems Engineering and Food Science, Zhejiang University, Hangzhou 310058, China; 3 Key Laboratory of Healthy Beverages, China National Light Industry, College of Food Science and Nutritional Engineering, China Agricultural University, Beijing 100083, China; 4 Fuli Institute of Food Science, Zhejiang University, Hangzhou 310058, China; 5 Innovation Center of Yangtze River Delta, Zhejiang University, Hangzhou 310058, China

**Keywords:** delivery system, in vitro digestion, ultrasound, protein–polysaccharide interaction, electrostatic interaction, Maillard reaction, stability, physicochemical property

## Abstract

In this study, we employed the ultrasound-prepared electrostatic complex and covalent conjugate of soy protein isolate (SPI) and citrus pectin (CP) to prepare β-carotene-loaded nanoemulsions. The in vitro digestion and storage stability of nanoemulsions stabilized by different types of emulsifiers were investigated and compared. Nanoemulsions stabilized by ultrasound-treated complex/conjugate showed the highest encapsulation efficiency; during gastric digestion, these nanoemulsions also demonstrated the smallest droplet sizes and the highest absolute values of zeta potential, indicating that both electrostatic complexation/covalent conjugation and ultrasound treatment could significantly improve the stability of the resulting nanoemulsions. In comparison, complexes were more beneficial for the controlled release of β-carotene; however, the conjugate-stabilized nanoemulsion showed an overall higher bioaccessibility. The results were also confirmed by optical micrographs. Furthermore, nanoemulsions stabilized by ultrasound-prepared complexes/conjugates exhibited the highest stability during 14-day storage at 25 °C. The results suggested that ultrasound-prepared SPI–CP complexes and conjugates had great application potential for the delivery of hydrophobic nutrients.

## 1. Introduction

As a provitamin A carotenoid abundant in colored vegetables, fruits and fungi [1], β-carotene is a powerful antioxidant that exhibits various health benefits, including immunity improvement, anti-aging, anticancer and anticardiovascular properties [2]. However, its relatively long hydrophobic chain and numerous conjugated double bonds generally result in a low water solubility and oral bioavailability, as well as the high vulnerability to light, heat and oxygen, greatly limiting its industrial uses [3,4]. In this case, researchers have made efforts to design various delivery systems to protect β-carotene during preservation and digestion [5]. Amongst those, protein-based O/W emulsions have attracted great attention.

Soy protein isolate is a low-cost food emulsifier with excellent nutritional and functional properties [6]. Emulsions prepared with SPI have proven useful in enhancing the chemical stability and water solubility of β-carotene [7,8]. However, changes in a variety of environmental factors including pH, temperature and ionic strength can readily lead to SPI denaturation and, in turn, instability of the resultant emulsions [9]. In this case, numerous attempts have been made to develop protein–polysaccharide binary combinations to improve the stability and functional properties of the emulsion [10,11,12,13,14,15].

The major approach to prepare protein–polysaccharide combinations is either by electrostatic attraction to form complexes, or by the Maillard reaction to form conjugates [16]. These binary biomacromolecule systems have demonstrated higher solubility, emulsifying properties and delivery stability than the mere protein [17,18,19]. As an acidic-soluble polysaccharide with numerous health benefits, pectin is the most commonly used stabilizer in protein-based acidic beverages [20]. Recently, complexes and conjugates of SPI and pectin have been prepared to stabilize O/W emulsions and encapsulate liposoluble nutrients for more desirable functional properties [21,22]. However, instability of these complex/conjugate-prepared emulsions often occurs due to diverse intermolecular interactions [23]. Moreover, given the extremely complicated structures of both biomacromolecules, the Maillard-type SPI–pectin conjugates usually have a very low degree of graft (DG), which, in turn, leads to very limited improvement in functionality [22]. Therefore, in recent years, ultrasound has increasingly been utilized in the preparation of protein–polysaccharide complexes/conjugates [24,25].

As an innovative food-processing technology, ultrasound has found utility in diverse industrial units to accelerate reactions and improve product qualities. In our previous studies [26,27], we employed ultrasound treatment in the electrostatic complexation and conjugation between SPI and citrus pectin (CP). The results showed that ultrasound treatment at 630 W for 10 min significantly increased the emulsifying activity index (EAI) and emulsifying stability index (ESI) by 44.1% and 24.68%, respectively. Furthermore, emulsions prepared using ultrasound-treated complexes were more homogeneous with smaller droplet size [26]. As for the SPI–CP conjugates, it was found that ultrasound treatment at 450 W for 45 min produced a DG of 24.06%, whereas the DG obtained from the traditional wet heating for 24 h was only 8.25%. In addition, the ultrasound-treated SPI–CP conjugates showed 147.59% and 102.76% higher EAI and ESI, respectively, than the conjugates prepared by traditional wet heating [27]. These results show that ultrasound-treated SPI–CP complexes/conjugates have great application potential for the delivery of hydrophobic nutrients.

Therefore, in this study, we applied ultrasound-prepared electrostatic complex and covalent conjugate of SPI and CP to prepare β-carotene-loaded nanoemulsions. The in vitro digestion and storage stability of nanoemulsions stabilized by different types of emulsifiers were investigated and compared. Despite the extensive studies establishing protein–polysaccharide complex/conjugate-based emulsions as delivery vehicles, few of them compared the performance of these two kinds of binary combinations during in vitro digestion. This study aims to compare the delivery behaviors and storage stabilities of nanoemulsions stabilized by (i) electrostatic complex and covalent conjugate; and (ii) binary systems prepared with and without ultrasound. The results of this study will further the understanding of delivery properties of protein–polysaccharide-based binary systems, as well as provide an innovative method for the production of these types of emulsifiers.

## 2. Materials and Methods

### 2.1. Materials

Defatted soy flour was purchased from Hengrui Food Co., Ltd. (Guangzhou, China). Citrus pectin (P9135, galacturonic acid ≥74.0%, dried weight), pepsin (P7125, ≥400 U/mg), pancreatin (P7545, 8 × USP), sodium urate and sodium lactate were obtained from Sigma-Aldrich Co., Ltd. (Shanghai, China). β-carotene (BR, 97%) and mucin (S12066, BR) were purchased from Yuanye Bio-Technology Co., Ltd. (Shanghai, China). Corn oil was purchased from Walmart (Hangzhou, China). Bile extracts (G913513, ≥45%) were purchased from Macklin Biochemical Co., Ltd. (Shanghai, China). All other reagents were of analytical grade and obtained from Sinopharm Chemical Reagent Co., Ltd. (Shanghai, China).

In this study, SPI (at pH 7.0) was set as the control. Abbreviations and explanations of all tested samples are specified in Table 1.

### 2.2. Preparation of SPI

The SPI was isolated according to our previous studies [22,26]. Briefly, defatted soy meals were dispersed in the deionized water (0.0549 μS/cm) at a 1:15 (*w*/*v*) ratio. The pH of the suspension was controlled at pH 9.0 for 1 h. Subsequently, the suspension was centrifuged at 5000 rpm and 4 °C for 30 min with a high-speed centrifuge (3K15, Sigma Laborzentrifugen GmbH, Osterode am Harz, Germany). The supernatant was collected and its pH value was adjusted to pH 4.5. Then, the precipitates were centrifuged at 5000 rpm and 4 °C for 30 min, and then reserved and redispersed in deionized water. The dispersion was neutralized and stirred overnight for full rehydration. The resulting dispersion was dialyzed at 4 °C for 48 h and lyophilized before use. A total protein content of (96.48 ± 0.36)% was obtained using the Kjeldahl method.

### 2.3. Preparation of SPI–CP Electrostatic Complex

The SPI–CP complex was prepared using optimized conditions reported in our previous study [26]. Briefly, SPI and CP were mixed in the deionized water at a ratio of 1:3 *w*/*w* (where the protein concentration was controlled at 5 mg/mL) and stirred at 4 °C and pH 3.5 for 2 h. The blend was then kept at 4 °C for 24 h to form the water-soluble electrostatic SPI–CP complex. As for the preparation of the ultrasound-treated SPI–CP complex (USPI–CP complex), a total volume of 20 mL of SPI/CP mixtures (1:3 *w*/*w*, pH 3.5) was added to a customized glass tube and sonicated for 10 min at an ultrasound intensity of 31.5 W/mL via a probe sonicator with a frequency of 22 kHz and a maximum operating power of 900 W (JY92-IIDN, Ningbo Scientz Biotechnology Co. Ltd., Ningbo, China). The selected ultrasound probe with a diameter of 1 cm and a temperature probe connected to the generator were immersed in the mixtures. The glass reactor was immersed in an ice-water bath to maintain the temperature of the dispersion at 25 °C (as monitored by the temperature probe).

### 2.4. Preparation of SPI–CP Conjugate

SPI–CP conjugates were prepared by wet heating and ultrasound-assisted Maillard reaction, respectively, using the optimized conditions reported in our previous work [27]. Briefly, an equal quantity of SPI and CP were mixed in deionized water and stirred for 2 h. Then, the pH of the suspension was adjusted to pH 10.0. After reacting at 70 °C for 24 h, the suspension was immediately cooled down in an ice-water bath to terminate the Maillard reaction. The resultant SPI–CP conjugates were then dialyzed at 4 °C for 48 h and lyophilized before use. As for the preparation of ultrasound-treated SPI–CP conjugates (USPI–CP conjugates), SPI and CP mixtures at a pH of 10.0 were added to a cylinder glass reactor and processed with a probe sonicator (JY92-IIDN, Ningbo Scientz Biotechnology Co. Ltd., Ningbo, China) at an ultrasound intensity of 4.5 W/mL for 45 min following similar procedures to those mentioned in Section 2.3. The temperature of the solution was kept at 70 °C during the Maillard reaction using a circulating water bath. After that, the suspension was immediately cooled down in an ice-water bath to terminate the reaction. The resultant SPI–CP conjugates were then dialyzed at 4 °C for 48 h and lyophilized before use.

### 2.5. Preparation of β-Carotene-Loaded Nanoemulsions

The oil phase was prepared by dissolving 0.1 g of β-carotene powder in 20 mL of corn oil. The dispersion was then heated in a water bath at 50 °C for 10 min. After that, the solution was kept at 25 °C and stirred for another 2 h under nitrogen protection. The protein concentration in the water phase was controlled at 5 mg/mL. The SPI and conjugate samples were dissolved in deionized water and stirred overnight at pH 7.0 and 4 °C, while the complex samples were prepared according to Section 2.3. The oil phase was homogenized using a homogenizer (Ultra-Turrax, Ika Works GmbH & Co. KG, Staufen, Germany) with the water phase at a ratio of 1:9 *v*/*v* at a speed of 10,000 rpm for 5 min to form coarse emulsions. Nanoemulsions were obtained using a high-pressure homogenizer (NanoGenizer, Will NanoBio Tech Co., Ltd., Suzhou, China) by further homogenizing the coarse emulsions at 13,000 psi (~89.6 MPa) for five cycles and then cooling them down to ambient temperature using an ice-water bath. The containers were quickly filled with nitrogen to avoid the oxidation of the β-carotene. The theoretical content of β-carotene in nanoemulsions was 0.05% *w*/*v*.

### 2.6. Measurement of Encapsulation Efficiency (EE)

The content of β-carotene in the nanoemulsions was determined according to Chen et al. [28]. Briefly, 1 mL of freshly prepared emulsion sample was blended with 3 mL of hexane or ethanol–hexane solution (1:2, *v*/*v*) to extract the unencapsulated or total β-carotene, respectively. Subsequently, the mixtures were centrifuged at 5000 rpm and 4 °C for 10 min using a high-speed centrifuge (HC-3018R, USTC Zonkia Scientific Instrument Co., Ltd., Hefei, China); the hexane phase was collected. The extraction process was repeated three times and the collected extracts were combined. Finally, the absorbance of the extracts at 450 nm was measured and the concentration of β-carotene was obtained based on a standard curve ($y=4.6121x-0.0058,\;R^{2}=0.9992$). The EE was calculated as follows:

(1) $$\mathrm{EE}\;\left(\%\right)=\left(1-\frac{c_{unencapsulated}}{c_{total,\;emulsion}}\right)\times 100\%$$

where $c_{unencapsulated}$ and $c_{total,\;emulsion}$ were the content of unencapsulated and total β-carotene in the emulsion, respectively.

### 2.7. In Vitro Simulated Digestion of β-Carotene Nanoemulsions

A three-phase in vitro digestion model [29] was used to evaluate the stability and release profiles of β-carotene nanoemulsions stabilized by different types of emulsifiers (i.e., SPI, SPI–CP complex, USPI–CP complex, SPI–CP conjugate, USPI–CP conjugate). First, freshly prepared nanoemulsions were placed into conical flasks and stood at 37 °C for 10 min (initial phase). Then, the heated nanoemulsions were mixed with an equal volume of simulated saliva fluid (SSF) preheated at 37 °C. The pH was adjusted to pH 6.8 and the mixtures were then incubated at 37 °C for 10 min with continuous shaking at 200 rpm (mouth phase). After that, the samples were mixed with preheated simulated gastric fluid (SGF) at a volume ratio of 1:1 and the pH of the mixtures was adjusted to pH 2.5 (stomach phase). Finally, after shaking at 37 °C for 2 h, the pH of the digested samples was immediately adjusted to pH 7.0 and an equal volume of preheated simulated intestinal fluid (SIF) was added (small intestine phase). The consequent mixtures were incubated at 37 °C for another 2 h and the pH of the mixtures was maintained at pH 7.0 during this process.

### 2.8. Measurement of Mean Size and Zeta Potential of Nanoemulsions

Nanoemulsions stabilized by different emulsifiers collected from different digestive phases were diluted in deionized water with a constant pH value before analysis. The mean size and zeta potential of nanoemulsions were detected using ZS Zetasizer Nano (Malvern Instrument Co., Ltd., Malvern, UK) following a previous protocol [26]. The samples were placed into the equipment and equilibrated at 25 °C for 2 min before each measurement.

### 2.9. Release Profiles of β-Carotene during In Vitro Simulated Digestion

The release profiles of β-carotene during in vitro digestion were recorded every 5 min during the initial phase and mouth phase, and every 30 min during the stomach phase and small intestine phase. The released β-carotene content was measured following the method mentioned in Section 2.6.

### 2.10. Optical Microscopy

The morphologies of the β-carotene nanoemulsions were observed using an optical microscope (UOP Co., Ltd., Chongqing, China; UB200i) equipped with a video camera. A drop of diluted nanoemulsion was placed on the glass slide, covered with a coverslip, and the images were captured under an objective lens of 40 magnifications.

### 2.11. Measurement of β-Carotene Bioaccessibility

After in vitro digestion, the digested samples were centrifuged at 10,000 rpm and 25 °C for 40 min using a high-speed centrifuge (HC-3018R, USTC Zonkia Scientific Instrument Co., Ltd., Hefei, China). The micelle phase (supernatant that is below the undigested oil phase) was collected and passed through a 0.45 μm syringe filter. Subsequently, the total content of β-carotene was analyzed following the method described in Section 2.6. The bioaccessibility of the β-carotene was calculated via Equation (2):

(2) $$\mathrm{Bioaccessibility}\;\left(\%\right)=\frac{c_{micelle}}{c_{total}}\times 100\%$$

where $c_{micelle}$ and $c_{total}$ represent the concentration of β-carotene in the micelle phase and in the original nanoemulsion, respectively.

### 2.12. Observations of Storage Stability of Nanoemulsions

The prepared β-carotene nanoemulsions stabilized by different types of emulsifiers were sealed in glass vials and stored at 25 °C for 14 days without direct light irradiation. The state of the nanoemulsions was observed and images were captured every 7 days.

### 2.13. Statistical Analysis

All of the experiments were conducted in triplicate and the results illustrated in the form of mean ± standard deviation (SD). Statistical analyses were performed using one-way ANOVA and Tukey’s test through SPSS 26.0 software (SPSS Inc., Chicago, IL, USA).

## 3. Results and Discussion

### 3.1. EE of β-Carotene Nanoemulsions Prepared Using Different Types of Emulsifiers

Table 2 shows the EE of the nanoemulsions stabilized by different emulsifiers. As can be seen, the EE of nanoemulsions stabilized by two complexes was significantly increased compared to those stabilized by the native SPI, which can be attributed to the elevated emulsifying properties as a result of SPI–CP electrostatic interactions. Ultrasound treatment could modify SPI and CP, and increase the contact frequencies between molecules due to the enhanced mass transfer; therefore, the electrostatic interactions and emulsifying properties of the complex were improved, leading to a higher EE [26]. However, the SPI–CP conjugate prepared through traditional wet heating produced a similar EE to the native SPI, which was ascribed to the low DG of this sample. The Maillard reaction between SPI and CP was difficult to achieve because of the complex structures of both biomacromolecules. As reported in our previous study [27], the DG of the SPI–CP conjugate obtained using the traditional method was only 8.25%; nevertheless, ultrasound could significantly increase the DG to 24.06% in a shorter period of time, possibly by disintegrating protein aggregates and exposing more reactive sites of both reactants. The greater the attachment of CP molecules, the greater the steric stabilization effects for the emulsion droplets; therefore, the nanoemulsions stabilized by USPI–CP conjugate exhibited a significantly higher EE. In conclusion, ultrasound treatment together with SPI–CP interactions contributed to a higher EE of the nanoemulsions, which may possibly result in a better protective effect for β-carotene [30].

### 3.2. Physicochemical Properties of β-Carotene Nanoemulsions during In Vitro Digestion

**Figure 1:**
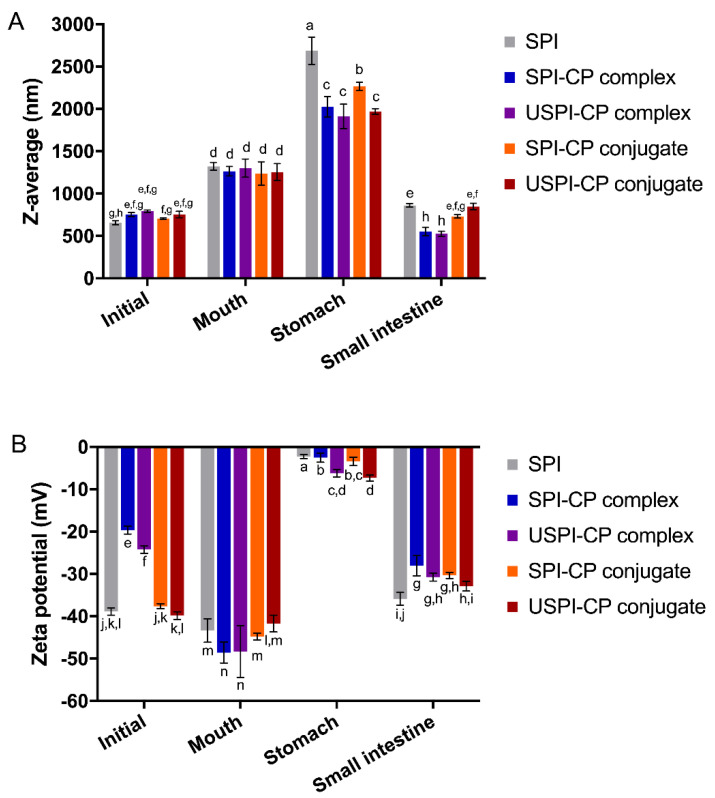
Changes of mean size (**A**) and zeta potential (**B**) of β-carotene nanoemulsions stabilized by different emulsifiers at different simulated in vitro digestion phases. Different letters (a–n) indicate significant differences as estimated by Duncan’s multiple range test (*p* < 0.05). Samples: SPI, soy protein isolate; SPI–CP complex, electrostatic complex formed between soy protein isolate and citrus pectin; USPI–CP complex, electrostatic complex formed between soy protein isolate and citrus pectin with ultrasound treatment; SPI–CP conjugate, Maillard-type conjugate formed between soy protein isolate and citrus pectin; USPI–CP conjugate, Maillard-type conjugate formed between soy protein isolate and citrus pectin with ultrasound treatment.

Figure 1 shows the mean size and zeta potential of nanoemulsions at different digestion stages. As can be seen from Figure 1A, in the initial phase, all nanoemulsions showed similar mean particle sizes ranging from 640 to 753 nm. The increased particle sizes in the mouth phase suggested the aggregation of droplets [30], which was attributed to the denaturation of SPI in the simulated saliva. In the stomach phase, all nanoemulsions showed a significantly larger mean size, indicating that severe droplet aggregations happened, which can lead to the release of β-carotene. This kind of instability of the nanoemulsions was due to the low pH value (pH 2.5) and changes in ionic strengths in SGF [31]. Furthermore, the pepsin hydrolysis of SPI molecules could further lead to the instability of wall layers [32,33]. The nanoemulsions stabilized by complexes and conjugates showed smaller droplets compared to those stabilized by the native SPI. This was ascribed to the steric hindrance effects that CP sustains, the attachment of which might block the way of pepsin to SPI. However, as the DG of SPI–CP conjugate was very low, the majority of this sample was actually the physical mixtures of SPI and CP. The weak interactions between SPI and CP made it less stable compared to other binary combinations; therefore, more droplet aggregates were present in nanoemulsions prepared using this sample, resulting in a larger droplet size. In the small intestine phase, the digestion of the oil droplets and SPI by pancreatin resulted in smaller mean droplet sizes for all samples [34].

The zeta potential of the nanoemulsions at different digestion stages is shown in Figure 1B. In the initial phase, the high absolute values of the zeta potential of SPI- and conjugate-stabilized nanoemulsions were due to the strong electronegativity of SPI molecules at pH 7.0. However, the pH of two complex samples was controlled at pH 3.5 to ensure the electrostatic interactions, where the CP molecules were negatively charged while SPI was positively charged; therefore, there was less net charge on the surface of the complexes. The relatively higher absolute value of zeta potential of USPI–CP complex-stabilized emulsions was due to the enhanced electrostatic interactions between SPI and CP under an ultrasonic field. In the mouth phase, the increased absolute values of the zeta potential of all samples were due to the negatively charged contents (e.g., mucin) in the simulated saliva. In the stomach phase, the pH (2.5) was near to the isoelectric point (pH 4.5) of SPI and pKa (pH 2.9) of CP [35], therefore, the net charges of all samples were near to zero. Another thing to note is that, although in theory, the SPI and CP molecules were positively charged in this stage, the zeta potential of the samples was still negative, which was possibly attributed to the electronegativity of the remaining mucin (with an isoelectric point at around pH 2.0) from the mouth phase. In addition to the protein denaturation under this extremely acidic condition, Guzey et al. [36] reported that the metal ions in SGF could also interrupt the electrostatic repulsion among droplets, leading to instability. The existence of CP molecules can be seen to be capable of mitigating these negative effects. As illustrated in Figure 1B, both complexation and conjugation with CP led to an increase in the absolute values of zeta potential. Furthermore, ultrasound treatment resulted in a greater increase for both samples, indicating a higher stability of the nanoemulsions. In the small intestine phase, most droplets were hydrolyzed into small colloidal particles, thus leading to the increased electronegativity [37]. In conclusion, the nanoemulsions stabilized using the ultrasound-prepared complex/conjugate showed the smallest droplet size and the highest absolute values of zeta potential in the stomach phase, indicating that protein–polysaccharide interactions and ultrasound treatment could significantly improve the emulsion stability at the gastric stage.

### 3.3. Release Profiles of β-Carotene during In Vitro Digestion of Nanoemulsions

**Figure 2:**
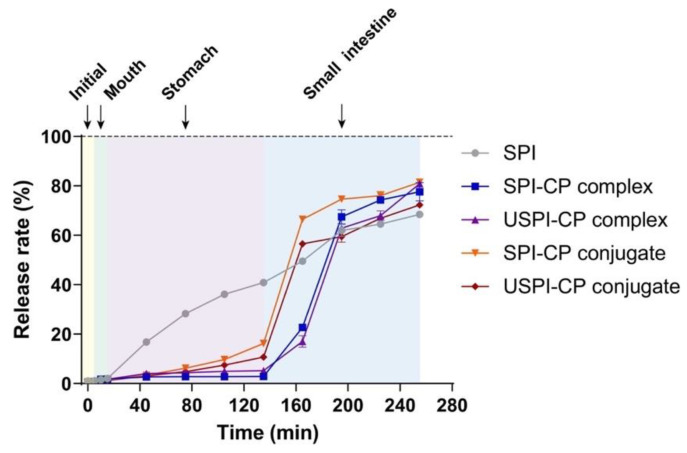
Release profiles of β-carotene from nanoemulsions stabilized by different emulsifiers during in vitro digestion. Samples: SPI, soy protein isolate; SPI–CP complex, electrostatic complex formed between soy protein isolate and citrus pectin; USPI–CP complex, electrostatic complex formed between soy protein isolate and citrus pectin with ultrasound treatment; SPI–CP conjugate, Maillard-type conjugate formed between soy protein isolate and citrus pectin; USPI–CP conjugate, Maillard-type conjugate formed between soy protein isolate and citrus pectin with ultrasound treatment.

Figure 2 illustrates the cumulative release curves of β-carotene during in vitro digestion. The cumulative release rate of β-carotene from the nanoemulsions stabilized by the native SPI increased smoothly in the whole process, with β-carotene cumulative release rates of 2.02%, 40.98% and 68.64% by the end of oral, gastric and intestinal digestion, respectively. On the contrary, by the end of the stomach phase, the β-carotene cumulative release rates of SPI–CP complex, USPI–CP complex, SPI–CP conjugates and USPI–CP conjugates were only 2.88%, 5.21%, 16.11% and 10.71%, respectively. After entering the intestinal digestion phase, the cumulative release rates for conjugate samples elevated significantly within 30 min, while the release rates for complexes significantly increased in 30–60 min; following this, all curves tended to flatten out. These results demonstrated the better protective effects of the complex and conjugate samples compared to the native SPI [38]. Comparing the complexes and conjugates, it was obvious that a slower release of β-carotene was achieved with complexes, possibly attributed to the higher CP content. Furthermore, ultrasound treatment can be seen to result in more desirable protective effects and controlled release for both complexes and conjugates, demonstrating that ultrasound could increase the stability of both binary systems. The final β-carotene cumulative release rates of SPI–CP complex, USPI–CP complex, SPI–CP conjugates and USPI–CP conjugates at 255 min were 77.05%, 80.34%, 80.93% and 72.49%, respectively. It was noteworthy that compared with other samples, USPI–CP conjugates exhibited a lower cumulative release rate, which was detrimental to the in vitro bioaccessibility of β-carotene. This might be attributed to the excessively strong protective effects of this sample. Compared to the electrostatic interaction, covalent bonding between SPI and CP provides a stronger binding force, which possibly protected the SPI from denaturation in the intestinal phase. The retention of these conjugates at the surface of the droplets would prevent contact between pancreatin lipase and the oil phase of the nanoemulsions, thus reducing β-carotene diffusion into the digestive fluid [17,39,40].

### 3.4. Morphology of β-Carotene Nanoemulsions during In Vitro Digestion

**Figure 3:**
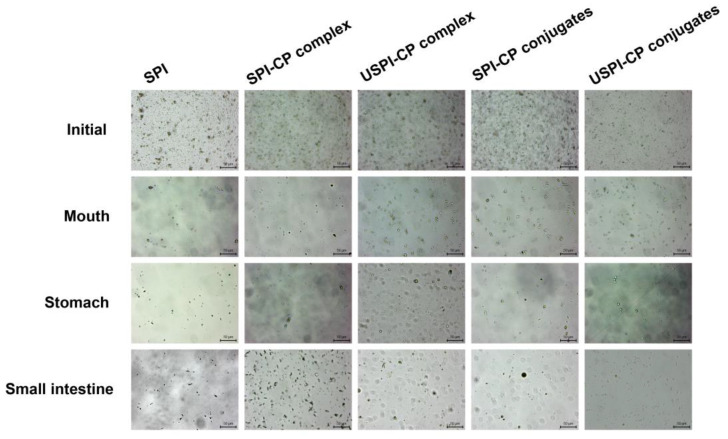
Optical micrographs of β-carotene nanoemulsions stabilized by different emulsifiers at different in vitro simulated digestion phases. Samples: SPI, soy protein isolate; SPI–CP complex, electrostatic complex formed between soy protein isolate and citrus pectin; USPI–CP complex, electrostatic complex formed between soy protein isolate and citrus pectin with ultrasound treatment; SPI–CP conjugate, Maillard-type conjugate formed between soy protein isolate and citrus pectin; USPI–CP conjugate, Maillard-type conjugate formed between soy protein isolate and citrus pectin with ultrasound treatment.

The morphology of the β-carotene nanoemulsions at different digestion phases was observed by optical microscope to provide an intuitive understanding of the above phenomena. As can be seen from Figure 3, the freshly prepared nanoemulsions with different emulsifiers all exhibited a homogeneous nature, with spherical droplets uniformly distributed. In the mouth phase, the SPI-stabilized nanoemulsions showed a significantly reduced number of droplets, indicating that the SPI structures were disrupted in the simulated saliva, leading to the oil-release phase. In contrast, the nanoemulsions stabilized by complexes and conjugates exhibited better stability, yet the droplets of these samples started to become irregular. In the stomach phase, the breakdown of certain numbers of droplets in the samples indicated that part of the encapsulated β-carotene was released. In comparison, most droplets in the SPI-stabilized samples can be seen to collapse, while there was still β-carotene embedded in the complex/conjugates samples, which demonstrated the more desirable protective effects of these binary systems. As mentioned above, the steric hindrance of CP molecules could effectively prevent SPI hydrolysis as well as the intermolecular interactions, which in combination led to higher stability. Nevertheless, the droplet sizes in complex/conjugate samples were significantly larger when compared to their initial state, possibly due to droplet aggregation and flocculation induced by the extreme pH and ionic strengths, as well as partial SPI hydrolysis [41]. In the small intestine phase, the majority of the droplets collapsed with large numbers of β-carotene released outside. However, as can be observed from Figure 3, there were still certain numbers of intact droplets left in nanoemulsions stabilized by USPI–CP conjugates, which could be a possible reason for the lower β-carotene cumulative release rate, as mentioned in Section 3.3. These results were in line with the previous analysis of changes in the mean size and zeta potential of nanoemulsions, as described in Section 3.2.

### 3.5. In Vitro Bioaccessibility of β-Carotene Nanoemulsions

**Figure 4:**
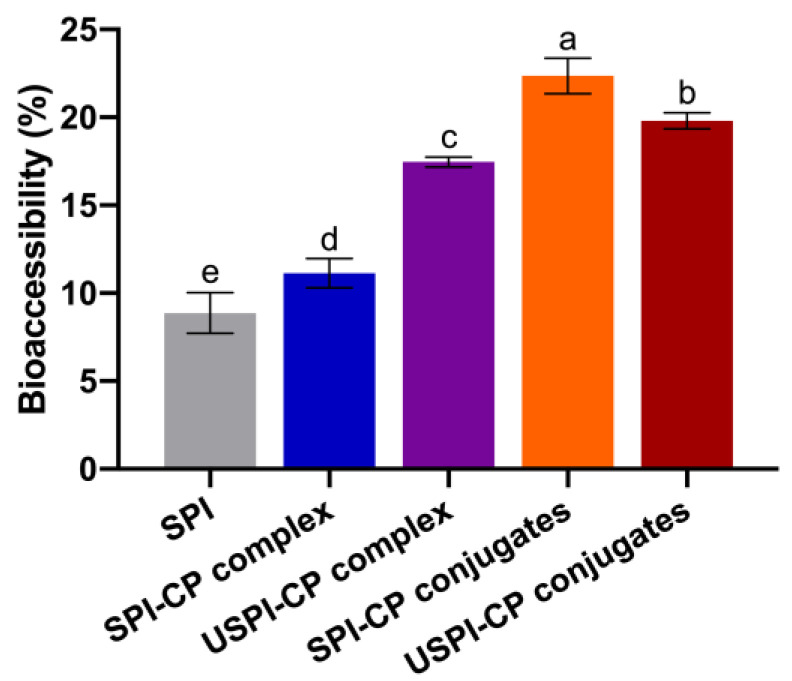
Bioaccessibility of β-carotene nanoemulsions stabilized by different emulsifiers. Different letters (a–e) indicate significant differences as estimated by Duncan’s multiple range test (*p* < 0.05). Samples: SPI, soy protein isolate; SPI–CP complex, electrostatic complex formed between soy protein isolate and citrus pectin; USPI–CP complex, electrostatic complex formed between soy protein isolate and citrus pectin with ultrasound treatment; SPI–CP conjugate, Maillard-type conjugate formed between soy protein isolate and citrus pectin; USPI–CP conjugate, Maillard-type conjugate formed between soy protein isolate and citrus pectin with ultrasound treatment.

Figure 4 depicts the bioaccessibility of β-carotene nanoemulsions stabilized by different emulsifiers. The acidic conditions of the gastric phase can readily lead to the protonation, isomerization and degradation of β-carotene, thus decreasing its bioaccessibility [42]. Therefore, the protective effects provided by the wall materials in the stomach phase are essential for high bioaccessibility. The bioaccessibility of the SPI-stabilized nanoemulsion was only 8.87%, indicating the poor stability of the native SPI during digestion. The bioaccessibility of SPI–CP complex- and USPI–CP complex-stabilized nanoemulsion was 11.14% and 17.46%, respectively, revealing that electrostatic complexation and ultrasound treatment produced a more stable protective layer for β-carotene. However, the highest bioaccessibility was achieved at 22.39% with the SPI–CP conjugates, while the USPI–CP conjugate-stabilized nanoemulsion showed a lower bioaccessibility of 19.80%. This phenomenon was in line with the results reported by Chen et al. [28], where the whey protein isolate–gum Acacia conjugates prepared under traditional dry heating exhibited a 52.30% higher bioaccessibility of β-carotene than the ultrasound-treated conjugates. As mentioned before, this might be attributed to the excessively strong protective effects provided by USPI–CP conjugates, which could prevent protein denaturation and the subsequent oil release. It was also noteworthy that the bioaccessibility of both complex-stabilized nanoemulsions was still relatively low compared to that of the conjugate-stabilized nanoemulsions. This could be due to the higher pectin content in the complex samples. As a dietary fiber, pectin was able to inhibit the formation of the micelle [43,44] and, in turn, decrease the in vitro bioaccessibility of β-carotene.

### 3.6. Storage Stability of β-Carotene Nanoemulsions

**Figure 5:**
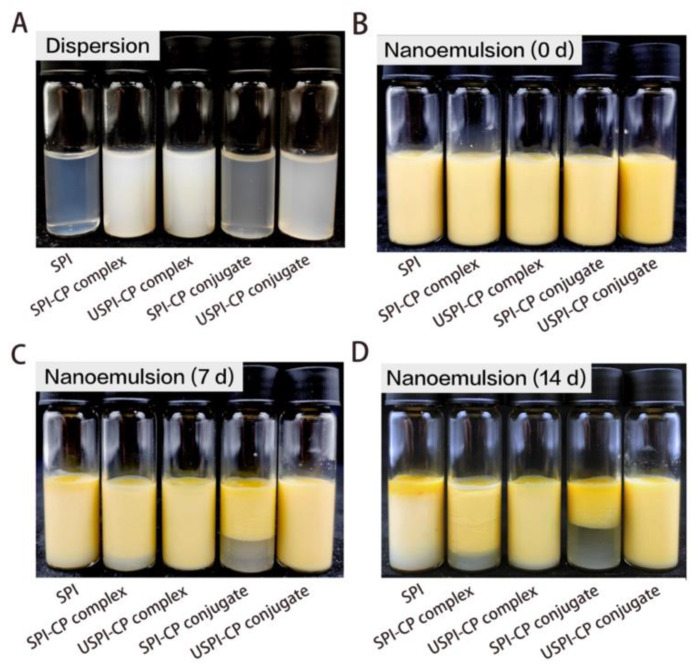
Images of original dispersions of SPI, SPI–CP complex, USPI–CP complex, SPI–CP conjugate and USPI–CP conjugate, respectively (**A**), and images of β-carotene nanoemulsions stabilized by different emulsifiers stored at 25 °C without direct light irradiation for 0 d (**B**), 7 d (**C**) and 14 d (**D**), respectively. Samples: SPI, soy protein isolate; SPI–CP complex, electrostatic complex formed between soy protein isolate and citrus pectin; USPI–CP complex, electrostatic complex formed between soy protein isolate and citrus pectin with ultrasound treatment; SPI–CP conjugate, Maillard-type conjugate formed between soy protein isolate and citrus pectin; USPI–CP conjugate, Maillard-type conjugate formed between soy protein isolate and citrus pectin with ultrasound treatment.

The appearance observations of the solutions and nanoemulsions prepared with different emulsifiers during 14-day storage are shown in Figure 5. As can be seen from Figure 5A, the addition of CP significantly increased the turbidity of the dispersion. The USPI–CP conjugates showed an obvious higher turbidity than SPI–CP conjugates due to the higher DG. Compared to the conjugate samples, the complex dispersions with a higher CP proportion exhibited higher turbidity. On the other hand, the freshly prepared β-carotene-loaded nanoemulsions with different emulsifiers showed an overall similar appearance (Figure 5B). However, after storage at 25 °C for 7 days, creaming and phase separation occurred in the nanoemulsions stabilized by SPI, SPI–CP complex and SPI–CP conjugate; with the storage time prolonged to 14 days, these emulsion instability phenomena became more aggravated. In contrast, the nanoemulsions prepared with the two ultrasound-treated binary combinations presented an overall stable nature—there were scarcely any changes in appearance after 7-day storage and very slight phase separation after 14-day storage. In this case, the two ultrasound-established binary systems are of higher practical value compared to the other samples.

## 4. Conclusions

This study investigated and compared the in vitro digestion and storage stability of β-carotene-loaded nanoemulsions prepared using SPI–CP complex/conjugate with and without ultrasound treatment. Nanoemulsions stabilized by USPI–CP complex/conjugate exhibited the highest EE. During in vitro digestion, the USPI–CP complex/conjugate showed the smallest droplet size and the highest absolute values of zeta potential in the stomach stage, indicating that electrostatic complexation/covalent conjugation and ultrasound treatment could significantly improve the stability of nanoemulsions. Compared with the conjugates, the complexes were more advantageous in terms of the controlled release of β-carotene. However, the conjugate-encapsulated β-carotene showed an overall higher bioaccessibility than the complexes. These results were confirmed by morphology observations using an optical microscope. Furthermore, the nanoemulsions with the ultrasound-prepared complex/conjugate showed an overall unchanged appearance after 14-day storage at 25 °C, while other samples exhibited severe creaming and phase separation phenomena. In conclusion, ultrasound-prepared SPI–CP complexes and conjugates have enormous potential in preparing emulsion-based systems for the delivery of hydrophobic nutrients, given their high stability and strong protective effects, as well as the high efficiency of the preparation procedures. In general, as the formation and preservation of electrostatic complexes have a strict requirement for pH, applications of the ultrasound-prepared complexes might be more suitable for acidic, liquid food matrices; applications of the conjugates are more flexible, which can be preserved in both solid and liquid states. This study advances the understanding of the delivery properties of protein–polysaccharide-based complexes and conjugates, as well as the merits that ultrasound provides when developing such binary systems.

## Figures and Tables

**Table 1:** Abbreviations and explanations of sample names.

Sample Names	Explanations
SPI	Soy protein isolate
SPI–CP complex	Electrostatic complex formed between SPI and CP
USPI–CP complex	Electrostatic complex formed between SPI and CP with ultrasound treatment
SPI–CP conjugate	Maillard-type conjugate formed between SPI and CP
USPI–CP conjugate	Maillard-type conjugate formed between SPI and CP with ultrasound treatment

**Table 2:** EE of β-carotene nanoemulsions prepared using different types of emulsifiers.

Emulsifiers	EE (%)
SPI	97.25 ± 0.02 ^d^
SPI–CP complex	98.08 ± 0.37 ^b,c^
USPI–CP complex	98.78 ± 0.08 ^a^
SPI–CP conjugate	97.59 ± 0.04 ^c,d^
USPI–CP conjugate	98.47 ± 0.21 ^a,b^

Note: values with different superscript letters (a–d) indicate significant differences as estimated by Duncan’s multiple range test (*p* < 0.05). Samples: SPI, soy protein isolate; SPI–CP complex, electrostatic complex formed between soy protein isolate and citrus pectin; USPI–CP complex, electrostatic complex formed between soy protein isolate and citrus pectin with ultrasound treatment; SPI–CP conjugate, Maillard-type conjugate formed between soy protein isolate and citrus pectin; USPI–CP conjugate, Maillard-type conjugate formed between soy protein isolate and citrus pectin with ultrasound treatment.

## Data Availability

The data presented in this study are available on request from the corresponding author. The data are not publicly available due to privacy reasons.
